# *Plasmodium matutinum* Causing Avian Malaria in Lovebirds (*Agapornis roseicollis*) Hosted in an Italian Zoo

**DOI:** 10.3390/microorganisms9071356

**Published:** 2021-06-23

**Authors:** Cristiano Cocumelli, Manuela Iurescia, Elena Lavinia Diaconu, Valentina Galietta, Caterina Raso, Carmela Buccella, Fiorentino Stravino, Francesco Grande, Letizia Fiorucci, Claudio De Liberato, Andrea Caprioli, Antonio Battisti

**Affiliations:** 1 Department of General Diagnostics, Istituto Zooprofilattico Sperimentale del Lazio e della Toscana “M. Aleandri”, 00178 Rome, Italy; cristiano.cocumelli@izslt.it (C.C.); manuela.iurescia@izslt.it (M.I.); elena.diaconu-esterno@izslt.it (E.L.D.); valentina.galietta-esterno@izslt.it (V.G.); caterina.raso-esterno@izslt.it (C.R.); carmela.buccella@izslt.it (C.B.); fiorentino.stravino@izslt.it (F.S.); claudio.deliberato@izslt.it (C.D.L.); antonio.battisti@izslt.it (A.B.); 2 Loro Parque Fundación, Avenida Loro Parque, Puerto de la Cruz, 38400 Tenerife, Spain; fragrande@alice.it; 3 Facultad de Veterinaria, Universidad de Las Palmas de Gran Canaria, Arucas, 35416 Las Palmas de Gran Canaria, Spain; letiziafiorucci@gmail.com

**Keywords:** *Agapornis* spp., Haemosporida, parrots, *Plasmodium*, Psittacidae, disease, zoo, captive birds

## Abstract

Avian malaria is a worldwide distributed, vector-born disease of birds caused by parasites of the order Haemosporida. There is a lack of knowledge about the presence and pathogenetic role of Haemosporida in Psittacidae. Here we report a case of avian malaria infection in lovebirds (*Agapornis roseicollis*), with the genetic characterization of the *Plasmodium* species involved. The birds were hosted in a zoo located in Italy, where avian malaria cases in African penguins (*Spheniscus demersus*) were already reported. Animals (*n* = 11) were submitted for necropsy after sudden death and were subjected to further analyses including histopathology, bacteriology, and PCR for the research of haemosporidians. Clinical history, gross lesions and histopathological observation of schizonts, together with positive PCR results for *Plasmodium* spp., demonstrated that avian malaria was the cause of death for one animal and the possible cause of death for the other nine. The sequences obtained were compared using BLAST and analyzed for similarity to sequences available at the MalAvi database. Genetic analyses demonstrated a 100% nucleotide identity to *Plasmodium matutinum* LINN1 for all the obtained sequences. To our knowledge, this is the first report describing avian malaria in lovebirds.

## 1. Introduction

Avian malaria is a mosquito-borne disease of birds caused by intracellular protozoa parasites of the order Haemosporida such as *Plasmodium*, *Haemoproteus,* and *Leucocytozoon* [1,2,3]. Avian *Plasmodium* parasites are transmitted by mosquitoes within the family *Culicidae*, with mosquitoes of the *Culex pipiens* complex probably being the main vectors in Europe [2,4,5,6,7]. The genus *Plasmodium* consists of more than 50 lineages worldwide distributed [2,3]. Identification of *Plasmodium* spp. has traditionally relied on morphological analyses of parasites seen in blood smears [8], but since the beginning of the 2000s, molecular characterization has led to the description of multiple parasites lineages, and these lineages have been gradually assigned to their respective morphospecies [3,9,10,11,12]. These parasites can cause severe health disorders in domestic, wild, and captive birds, sometimes leading to lethal malaria [8,11,13,14,15,16,17,18,19,20]. In particular, they are responsible of significant mortality in captive birds (zoos or private collections), when captive birds of exotic species are exposed to local *Plasmodium* strains [17,18,21]. A plausible reason for the susceptibility to the disease observed in some captive bird species is the lack of selective pressure for hosts to evolve appropriate innate immune responses to these parasites [22]. Another possible explication is that the stress associated with captivity may increase the immunological susceptibility of individuals, thus increasing parasite load, or even reduce their capacity to avoid the vectors commonly present in zoos, thus increasing parasite prevalence [23].

In Italy, some earlier and more recent studies reported the presence of different *Plasmodium* species in wild birds without evidence of disease, including *P. matutinum*, *P. giovannolai* [24,25], *P. relictum*, *P. vaughani* [26], *P. circonflexus,* and *P. polare* [27]. In another study on blood-fed mosquitoes from Northern Italy, Martínez-de la Puente et al. [7] reported the presence of six *Plasmodium* lineages in different mosquito species, including *P. relictum* and *P. vaughani*. More recently, Iurescia et al. [28] reported avian malaria (from *P. matutinum)* in dead African penguins (*Spheniscus demersus*) from two Italian zoos located in Central Italy and in *Culex pipiens*. In the same study *P. vaughani* was also detected in *Culex pipiens* [28].

Parrot species in the wild are infrequently reported to be infected by haemosporidians [23,29,30]. Different studies demonstrated that captive parrots of several species can become infected by these parasites [23,29,31,32,33,34,35], with some report of fatal infections [1,22,30,36,37]. In particular, in Europe, *Haemoproteus minutus* has been found to be responsible for several outbreaks in aviaries hosting parrot species from Australasia and South America [1,37]. Regarding specifically *Plasmodium* species, Belo et al. [33] performed a cross-sectional survey to estimate the occurrence of malaria infection among 127 captive psittacine birds from three zoological gardens in Brazil, demonstrating an overall prevalence of 36%. The sequence analyses of 10 isolates indicated a potential occurrence of four distinct *Plasmodium* lineages. Baron et al. [34] found at least four different strains of *Plasmodium spp*. circulating in the budgerigar populations of New Zealand’s North Island, and Verwey et al. [22] reported the death of captive psittacine species (*Calyptorhynchus funereus* and *Calyptorhynchus lathami*) due to *Plasmodium* sp. in Australia.

Here we describe a case of avian malaria infection in lovebirds (*Agapornis roseicollis*) kept in a zoo located in Central Italy, along with the genetic characterization of the *Plasmodium* species involved.

## 2. Materials and Methods

### 2.1. Animal Collection

Between August and October 2017, 11 lovebirds (*A. roseicollis*) died in a zoo located in Central Italy (Ecoregion: Mediterranean forests, woodlands, and scrub). The zoo occupies about 99 acres (40 hectares) and hosts about 350 animals of 36 different species (mammals, birds, and reptiles). During the study period, the zoo hosted about 150 Psittacidae, including around 80 *Agapornis* spp. and other avian species native to Australasia, South America and Africa. All the lovebirds involved in the study were acquired in 2016 at about six months of age from two different Italian breeder suppliers. Before admission, the birds were subjected to a period of standard quarantine and then moved to an outdoor aviary together with lesser flamingos (*Phoeniconaias minor*) and Australian pelicans (*Pelecanus conspicillatus*). Clinical history was of sudden death without any previous evident symptom. The birds were sent to the Istituto Zooprofilattico Sperimentale del Lazio e della Toscana “M. Aleandri” (IZSLT) for necropsy examination and to establish the causes of death. The birds were sent in two different occasions; the first submission comprised two fresh subjects who died in October 2017, while the second submission comprised a group of nine frozen lovebirds died during the month of August 2017, only retrospectively sent.

### 2.2. Pathology, Histopathology, and Bacteriology

All the 11 animals received were subjected to a necropsy examination. Samples of multiple organs from the two animals of the first submission were collected and fixed in 10% neutral buffered formalin for histopathological examination following standard procedures. Due to freezing and the poor post-mortem conservation status, histopathology was not performed on the nine animals of the second group. Whenever considered pertinent, tissue samples for additional standard bacteriological analyses were also collected.

### 2.3. Haemosporidians Molecular Identification and Characterization

For PCR analysis, liver and spleen (target organs for the diagnosis of avian malaria) from the two animals of the first group were collected and tested, separately. Because of the poor post-mortem conservation status, PCR was performed only on a pool of organs (liver and spleen) from three of the nine subjects of the second group (three livers and three spleens pooled together).

Total DNA was extracted from the organs using an automated system (QIAsymphony^®^ SP Qiagen) with the DSP Virus/Pathogen Mini Kit, following the manufacturer’s instructions.

Extracted DNA was subjected to a simplex PCR assay for the detection of the three haemosporidian genera (*Plasmodium*, *Haemoproteus* and *Leucocytozoon*), using as a target the partial mitochondrial conserved region of the cytochrome b gene (cyt b). The PCR was performed with the primers HaemNFI (5′-catatattaagagaaitatggag-3′) and HaemNR3 (5′-atagaaagataagaaataccattc-3′) described by Hellgren et al. [38], using the following protocol: Master Mix was prepared with 5 μL of distilled water, 12.5 μL of Platinum™ Hot Start PCR Master Mix 2X (Invitrogen), 1.25 μL of each M13-tailed primer (10 pMoL/μL), with a final volume of 20 μL, then 5 μL of the template was added.

PCR started with an initial denaturation step of 3 min at 95 °C, followed by 35 cycles of 15 s at 95 °C, 15 s at 50 °C, 5 s at 72 °C, and a final extension at 72 °C for 3 min. Negative and positive controls for both DNA extraction and PCR reaction processes were also included. For sequencing, positive PCR-products (570 bp) were purified using an enzymatic cleanup ExoSAP-IT™ kit. Amplicons were Sanger sequenced on a 3500 Series Genetic Analyzer with BigDye Terminator chemistry (Applied Biosystems, Foster City, CA, USA) using the same primers. Sequence data analysis and trimming was performed using the CLC DNA workbench^®^ software version 5.7.1 (CLC Bio-Qiagen, Aarhus, Denmark). The resulting sequences were examined for the presence of overlapping peaks to exclude potential co-infections and compared using BLAST (online version, blastn algorithm, https://blast.ncbi.nlm.nih.gov/Blast.cgi, accessed on 30 April 2021) [39]. The cyt b sequences were also compared for similarity to sequences available at the MalAvi database (http://mbio-serv2.mbioekol.lu.se/Malavi, accessed on 30 April 2021) [40].

## 3. Results

### 3.1. Pathology, Histopathology, and Bacteriology

**Figure 1:**
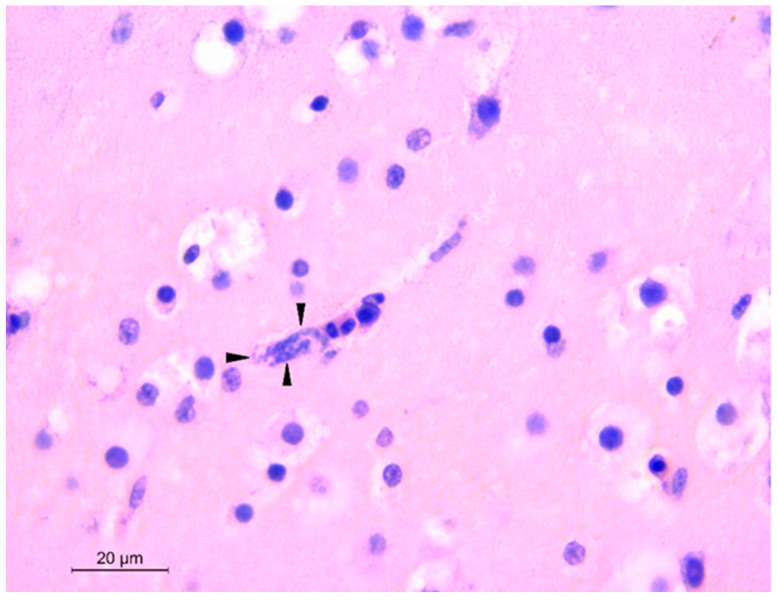
Brain. *Agapornis roseicollis*. Histological section of brain with an intracapillary schizonts (arrows head) containing multiple small basophilic structures (merozoites). Hematoxylin-eosin.

Following necropsy and histopathology, one bird of the first submission was diagnosed with a non-avian malaria cause of death. This animal showed typical lesions referable to visceral gout. The second bird had splenomegaly, miliary necrotic foci on the liver, and a mild brain hyperemia. At histopathology, round to elongated structures (schizonts) were observed inside the cytoplasm of endothelial or reticuloendothelial cells of the liver and spleen. In the brain, schizonts were observed inside the capillaries. Schizonts were 20 to 80 μm in diameter and were filled with many 1–3 μm basophilic merozoites (Figure 1). No intraerythrocytic parasites were detected at histopathological observation. The nine animals of the second submission presented noticeable splenomegaly and hepatomegaly. Standard bacteriological analyses performed on organs (liver, lung, brain, intestine) of the second animal of the first submission gave negative results. The other animals were not tested either because of the poor state of conservation, or because presenting lesions not suggestive of bacterial infections (the animal with visceral gout).

### 3.2. Haemosporidians Identification and Characterization

The spleen and liver of the animal showing lesions referable to visceral gout tested PCR negative. All the other examined samples (n = 3 samples: spleen and liver of the second animal of the first group and pooled livers and spleens of three animals of the second group) tested positive for *Plasmodium* spp. Blast analysis demonstrated that all sequences were 100% identical one to each other and showed a 100% nucleotide identity to *P. matutinum* lineage LINN1. Analyzing the chromatograms, no evidence of double peaks (suggestive of mixed infections) was detected.

The three obtained sequences were submitted to the European Nucleotide Archive (ENA, http://www.ebi.ac.uk/ena, accessed on 30 April 2021) under the study accession number PRJEB44497.

## 4. Discussion

Psittaciformes are one of the most endangered groups of birds worldwide [29,37]. The role of zoos in conservation programs has increased significantly in last decades, and the health of captive animals is essential to guarantee success of such programs [35]. It is known that the same lineages of malaria parasites can infect birds belonging to different species, families, and even orders, but the knowledge about the prevalence and diversity of *Plasmodium* spp. and relative haemosporidians in zoo bird collections is at present insufficient [35].

In this study the death of 11 lovebirds (*A. roseicollis*) hosted in an Italian zoo was investigated. Histopathology and/or haemosporidians PCR analysis were performed only on a limited number of the submitted birds due to the poor state of conservation. However, clinical history of sudden death, gross lesions, and histopathological observation of schizonts, together with positive PCR results and absence of other evident causes of death, demonstrated that avian malaria was the most probable cause of death for one animal and the possible cause of death for other nine.

Hemoprotozoa are rarely reported as cause of death in parrots [1,22,30,36,37], and to our knowledge, this is the first report of avian malaria in *Agapornis* genus (parrots native to Africa), and the first report of *P. matutinum* LINN1 infection in parrots.

Gross pathological and histopathological findings were analogue to those described by other authors in other avian species, as well as in parrots [1,22,41]. Sudden death is also reported to be associated to Haemosporida infections in birds, particularly in the late stage of the infection when intracellular parasites determine obstruction of small vessels causing capillary ischemia, particularly in the brain [41]. The absence of intraerythrocytic forms of the parasite in the examined organs, giving the low sensitivity of the histopathology, could be due to the chronic stage of the infection [41], when the presence of the parasite would be reduced in terms of duration and quantity. Ortiz-Catedral et al. [37] suggest that the low presence of intraerythrocytic parasites could represent the expression of an abortive stage in the life cycle, while other authors report mortality in parrots associated with an intraerythrocytic stage of the infection [32,42]. Further studies are needed to clarify these aspects.

On the genomic site, *P. matutinum* LINN1 was already found to be associated to deaths in wild birds kept in zoos [12,28,43], although this is the first time it was detected in parrots. In particular, our research group previously demonstrated that *P.* *matutinum* was the etiological agent responsible for the deaths of African penguins (*Spheniscus demersus*) kept in the same zoo where the lovebirds were housed, and in that case *Culex pipiens* was the most probable vector of the infection [28]. Our results would unequivocally confirm the pathogenicity of *P. matutinum* LINN1, at least in some captive birds. This is of concern since demonstrates that both captive and eventually wild exotic invading species of parrots in Europe could be threatened by this lineage. Indeed, differences regarding the virulence of different *Plasmodium* lineages in different species have been suggested and need to be further investigated [12,44].

Interestingly, no other bird species beside penguins and lovebirds died because of avian malaria in the zoo at the time of the study, including parrot species native to Australasia and South America, and previously reported to be susceptible to the disease [1,34,37].

In order to clarify if the infection was present also in other birds of the same or related species at a subclinical level, to analyze blood samples from live birds would have been useful. Unfortunately, at the time of the study blood sampling was not possible due to management issues and welfare concerns.

Further studies are needed to understand if certain avian species at the zoo are more resistant than others to the infection and/or to the disease, if there is a preference in host feeding habits of the vectors, and to better understand the complex parasite/host/environment interactions.

This study also confirms that zoos maintaining captive birds in temperate areas where vectors are abundant should be well aware of the risks of avian malaria and should increase the effort to prevent the infection, in particular during periods when the number of vectors is higher [28,35]. Indeed, it is difficult to avoid haemosporidian parasite transmission in zoos, and the possible preventing measures have relevant management implications. For some species, it is possible to reduce the infection rate by treating susceptible animals with antimalarial drugs [17], or even by administering a diet containing food that provides anti-parasitic secondary metabolites that could be used as self-medication to reduce parasite load [23]. Other possible strategies aim at reducing the number of mosquitoes around the facilities, such as the use of mosquitoes traps, the use of fans, water vents, the spraying of the nests surroundings with neem oil or other mosquitoes repellents and the treating of standing waters with larvicides [17,28,35]. In some circumstances, it may be beneficial for species that are unlikely to have been exposed to mosquito vectors in the wild to be isolated from vectors during periods of high vector activity [22]. In this case, unfortunately, parrots were hosted in an outdoor aviary and were not subjected to any preventing measure. However, it has to be noticed that deaths in penguins occurred in the same zoo even if periodically treated with chloroquine phosphate and primaquine [28].

## 5. Conclusions

In conclusion, to our knowledge, this is the first report describing avian malaria in *Agapornis* genus (parrots native to Africa) corroborated with description of anatomo-histopathological lesions and identification of the *Plasmodium* species (*P. matutinum* LINN1) involved. It is interesting to note that the infection in lovebirds was detected in the same zoo with simultaneous mortality in African penguins due to avian malaria caused by *P. matutinum* LINN1, with *Culex pipiens* being demonstrated the most probable vector infecting the penguins [28]. These results confirm the importance to adopt effective prevention measures to avoid or reduce the transmission of blood parasites among captive birds and to put every effort to prevent avian malaria outbreaks. Further studies will be useful to assess the prevalence of the infection in the zoo by possibly evaluating the presence of the parasites in the blood of live birds, and to better understand the eco-epidemiology of the infection in parrots and other captive birds. This further information will be of utmost importance to improve effective control and prevention strategies.

## Data Availability

The sequences presented in this study are openly available in the European Nucleotide Archive (ENA) under the study accession number PRJEB44497.
